# Interpretable vision mamba for SAR images classification

**DOI:** 10.3389/fnbot.2026.1753927

**Published:** 2026-04-23

**Authors:** Kaiming Zou, Ningbo He, Leijun Yao, Jialin Li, Yongfeng Wang

**Affiliations:** 1 School of Geophysics, Chengdu University of Technology, Chengdu, China; 2 China Aneng Group Third Engineering Bureau Co., Ltd., Chengdu, China; 3 Xi'an Jiuzhi-Tech. Co., Ltd., China

**Keywords:** class activation mapping, explainable artificial intelligence, image classification, mamba, SAR ATR

## Abstract

Deep learning techniques have been successfully applied to object classification in Synthetic Aperture Radar (SAR) images, achieving remarkable performance. However, the current Transformer architecture still faces two limitations in SAR object classification: (1) Transformers occupies substantial GPU memory during training, which hinders large-scale model deployment and hyperparameter optimization. (2) the vectorization of image patches destroys spatial information among pixels, thus the represented features are semantic-agnostic. In this paper, we propose a lightweight and interpretable vision mamba (IVim) whose feature maps from deep Mamba blocks are visually understandable. IVim consists of two modules: Token Semantic Re-allocation (TSR) and Token Attention Selection (TSA). TSR re-allocates the semantics to feature maps by optimizing a novel loss function for an extra convolutional layer. TAS selects the most discriminative attention map by evaluating the quantity of semantics in attention matrices of different Mamba layer. Finally, we provide a coupling strategy to form a saliency heatmap to visually show the interpretability of IVim by merging feature map and attention map. Experimental results demonstrate that IVim can re-allocate semantics to deep features and achieve better classification performance compared to its counterparts by reducing 67.8% parameters.

## 1 Introduction

With the all-day and all-weather perception capability (Zhang et al., 2024), Synthetic Aperture Radar (SAR) is widely applied in various remote sensing tasks, like topographic survey (Wang M. et al., 2022), disaster rescue (Yang et al., 2025), electronic reconnaissance (Jinke et al., 2023; Sun et al., 2022), etc. Meanwhile, numerous deep-learning models have demonstrated remarkable success in general computer vision tasks (e.g., classification, detection, segmentation), which naturally inspires transferring them from optical image to SAR image. One of the most important vision task in SAR image processing is target classification because other tasks [e.g.,fine-grained classification (Xiong et al., 2024; Zheng et al., 2024; Xu and Lang, 2020), small-target detection (Feng et al., 2022; Liu P. et al., 2024; Chang et al., 2025), change detection (Costa et al., 2024; Zhang et al., 2022), despeckling (Hu et al., 2024; Lin et al., 2022)] usually adopt classification models as the backbones.

Convolutional Neural Network (CNN) is the first successfully-applied deep-learning architecture in SAR target classification. (Wang et al. 2018) firstly utilized CNN to implement SAR target recognition and obtained better accuracy than SVM. (Min et al. 2019) proposed a gradually distilled CNN with a small structure and high calculation efficiency for SAR target recognition. (Li et al. 2022) propose a mutiscale CNN for SAR automatic target recognition (ATR) by fusing multiscale feature maps obtained from different layers to enhance the feature description ability. However, CNN can only capture small-range feature dependency but almost unable to capture long-range feature dependency due to its convolution kernel's limited perception field, so CNN suffers from “feature bottleneck”. To better capture long-range feature dependency, (Vaswani et al. 2017) proposed a multi-head self-attention architecture, termed Transformer. Owing to multi-head self-attention mechanism, Transformer is able to capture long-range feature effectively, and numerous Transformer-based SAR target classification modes are proposed, like (Xiong et al. 2025); (Dai et al. 2025); (Wang C. et al. 2022); (Shang et al. 2024). However, multi-head self-attention mechanism make Transformer-based models commonly face two challenges:(1) It is low-efficient to train Transformer-based because of slow speed of inference. (2) A large amount of GPU memory is occupied during Transformer-based models training process, which is a challenge for hardwares. In view of CNN and Transformer's above limitations, it is imperative to have a novel deep-learning architecture with ability to capture long-range feature dependency like Transformer but with much faster inference speed and less GPU memory occupation.

**Figure 1:**
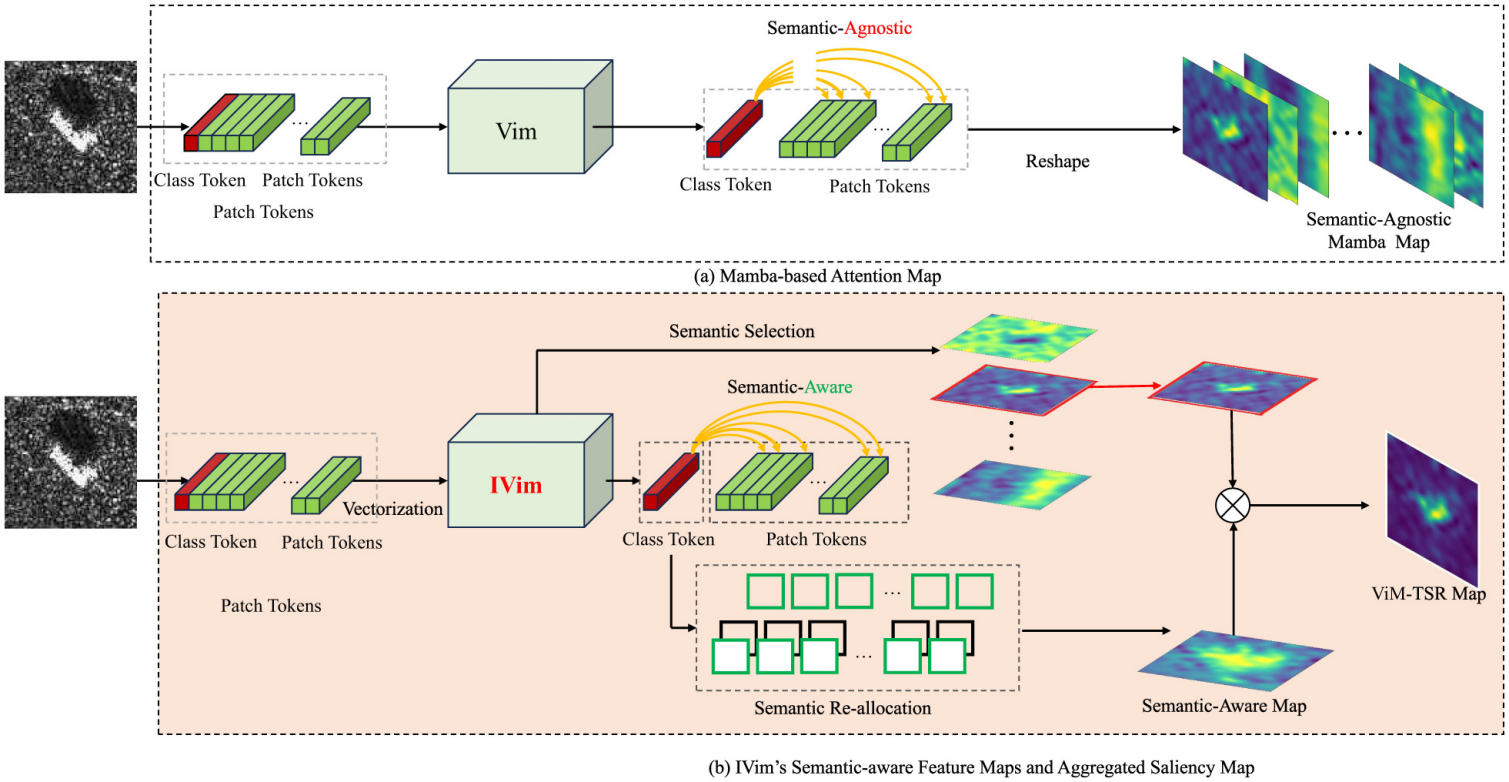
The comparison of representation mechanism of CNN feature maps, Vim's feature maps, IVim's feature maps.

To compensate for the above limitations, Recently, Gu et al. proposed a Linear-Time Sequence Modeling with Selective State Spaces (Mamba), a lightweight deep neural network based on state space model (SSM). Mamba is originally proposed for NLP tasks and demonstrates comparative performance to Transformers. Subsequently, Zhu et al. firstly transfer Mamba to vision tasks, termed as Visual Mamba (Vim). Compared with ViT, Vim is a lightweight model with linear computational complexity to input image dimension, saving 86.8.2% of GPU memory in comparsion to counterpart Transformer models. (Xu et al. 2025) proposed MambaShadowDet for moving target shadow detection. (Tu et al. 2025) proposed a Mamba unsupervised domain adaptation model for SAR ship detection. (Pan et al. 2025) proposed a multiscale and multibrmanch Mamba for SAR-assisted optical image thick cloud removal. (Li et al. 2025) proposed a Mamba-based joint semantic segmentation network for optical and SAR images. However, Mamba-based models lack interpretability in comparison to CNN since the feature maps represented by Vim are usually semantic-agnostic. It is because the vectorization of 2-D patches destroys the spatial information between image pixels. In this case, the feature maps exhibit no semantics. It is worth noting that existing interpretability methods for deep learning models typically provide an intuitive understanding of model mechanisms by visualizing the associations between deep feature representations and model predictions, basically including perturbation-based explanations and activation-based explanations. Perturbation-based methods (Kumar Singh and Jae Lee, 2017) generally design a mask to occlude or blur some pixels of the input image, and reveal the model's prediction mechanism by comparing the differences of predictions before and after the perturbation. However, perturbation-based explanations usually require multiple Monte Carlo trials to offset the bias induced by perturbation randomness, significantly decreasing efficiency when applied to large-scale models. Consequently, activation-based explanations ave become the dominant approach that produce saliency heatmaps by aggregatiing deep activation maps weightedly. Activation-based explanations mainly consist of class activation mapping (CAM) (Zhou et al., 2016) as well as its variants (Selvaraju et al., 2017; Ramaswamy et al., 2020; Wang et al., 2020; Feng et al., 2024, 2023). Nevertheless, they are still unable to directly visualize deep features representation mechanism of Vim, as shown in Figure 1. This lack of interpretability severely restricts the development of Mamba-based models in some sensitive and risky task, like disaster rescue, electronic reconnaissance, as mentioned in Section 1.

In this paper, we propose an interpretable Vision Mamba (IVim) architecture especially for SAR image classification. IVim involves two intertwined network branches: Token Semantic Reassignment (TSR) and Token Attention Selection (TAS). TSR re-allocates semantics for the patch tokens to generate a semantic-aware feature map by cascading an modified MLP classifier after the last Vim block and retrain the model with a new loss function. TAS selects the most discriminative attention map by referring to the confidence drop of the filter image masked by each attention map. Finally, attention map and feature map are merged as an interpretation heatmap revealing the most discriminative regions for IVim's classification.

The remainder of this paper is organized as follows. Section 2 introduces the basic theory of Mamba and reviews several Mamba models in SAR target classification. Section 3 elaborates the methodology of IVim. Section 4 provides experimental results from various perspectives to demonstrate the superiority of IVim compared to other models. Section 5 discusses the experimental results and clarifies the confusion.

## 2 Related work

In addition to object-level classification in single SAR images, broader remote sensing tasks such as scene classification and multitemporal analysis have also seen significant advancements, which further contextualize the motivation for interpretable and efficient architectures like IVim. Scene classification aims to assign a semantic label (e.g., residential, agricultural, industrial) to an entire image patch, requiring the model to capture global contextual relationships and spatial layouts. In contrast, multitemporal classification focuses on analyzing changes or consistent patterns across images acquired at different times, demanding robust temporal feature integration and sensitivity to subtle variations over time.

Recent studies have introduced sophisticated architectures to address these challenges. For instance, (Zhao et al. 2024) proposed a Gradient-guided Multi-scale Focal Attention Network for remote sensing scene classification, which leverages gradient information to adaptively focus on discriminative regions across multiple scales, enhancing model interpretability and accuracy in complex scenes. Similarly, (Wang et al. 2023a) developed a Multistage Self-Guided Separation Network that progressively disentangles semantic features from cluttered backgrounds, improving scene classification performance by emphasizing task-relevant visual cues. In the realm of multitemporal analysis, (Zhao et al. 2025) incorporated difference information into a curriculum learning framework, enabling more effective feature learning for classifying land cover changes over time.

While these methods have advanced scene understanding and temporal analysis, they predominantly rely on attention-based or CNN-based backbones, which inherit limitations such as high computational cost and limited interpretability of deep feature representations—issues similarly faced in SAR object classification. The proposed IVim architecture, with its efficient long-range dependency modeling and built-in interpretability modules (TSR and TAS), offers a promising alternative not only for SAR target classification but also for broader remote sensing tasks that require both efficiency and transparency. By reallocating semantics to feature maps and selecting semantically meaningful attention regions, IVim aligns with the growing need for models that are not only accurate but also explainable, particularly in critical applications like disaster monitoring, land use analysis, and environmental change detection where understanding model decisions is as important as performance.

### 2.1 State space model mechanism

**State space models for long sequence modeling** (Gu et al. 2021) propose a Structured State-Space Sequence (S4) model, a novel alternative to CNNs or Transformers, to model the long-range dependency. The advantageous property of linearly scaling in sequence length stimulates significant research interests. (Wang J. et al. 2022) introduce a Bidirectional Gated SSM architecture that replicates BERT's performance without attention mechanisms. Tan et al. (2019) present the S5 layer, incorporating MIMO SSM formulations and efficient parallel scan operations into the S4 framework. (Fu et al. 2022) design the H3 SSM layer, substantially narrowing the performance gap between SSMs and Transformer attention in language modeling tasks. (Mehta et al. 2022) develop the Gated State Space layer to enhance the expressivity of S4 through additional gating units. Recently, (Gu and Dao 2023) propose a data-dependent SSM layer and establish Mamba, *i.e*., a generic language model backbone that surpasses Transformers across scales on large real-world datasets while preserving linear sequence-length scaling.

The underlying architecture of Structured State Space (S4) and Mamba models is derived from a continuous framework. The core of this framework is a state-space system that transforms an input function *x*(*t*) ∈ ℝ into an output *y*(*t*) ∈ ℝ by a hidden state *h*(*t*) ∈ ℝ^*N*^. This system contains **A** ∈ ℝ^*N*×*N*^ as the evolution matrix and **B** ∈ ℝ^*N*×1^, **C** ∈ ℝ^1 × *N*^ as the projection parameters. Next, we will elaborate the mechanism of Mamba.


$$\begin{array}{l} h^{\prime }\left(t\right)=\text{A}h\left(t\right)+\text{B}x\left(t\right),\\ y^{\prime }\left(t\right)=\text{C}h\left(t\right). \end{array}$$ (1)

The S4 and Mamba are the discrete versions of above continuous system, so they further have a scaling parameter Δ to transform the continuous **A**, **B** to discrete $\bar{\text{A}}$, $\bar{\text{B}}$. by zero-order hold (ZOH), which is formulated as Equation 2


$$\begin{array}{l} \bar{\text{A}}=\exp\left(\text{Δ}\text{A}\right),\\ \bar{\text{B}}={\left(\text{Δ}\text{A}\right)}^{-1}\left(\exp\left(\text{Δ}\text{A}\right)-\text{I}\right)\text{Δ}\text{B}. \end{array}$$ (2)

After the discretization of $\bar{\text{A}}$, $\bar{\text{B}}$, the discretized version of Equation 1 using a step size Δ can be rewritten as Equation 3:


$$\begin{array}{l} h_{t}=\bar{\text{A}}h_{t-1}+\bar{\text{B}}x_{t},\\ y_{t}=\text{C}h_{t}. \end{array}$$ (3)

Finally, the output is given by a global convolution as


$$\begin{array}{l} \bar{\text{k}}=\left(\text{C}\bar{\text{B}},\text{C}\bar{\text{A}}\bar{\text{B}},\ldots ,\text{C}{\bar{\text{A}}}^{\left(M-1\right)}\bar{\text{B}}\right),\\ y=x*\bar{\text{k}} \end{array}$$ (4)

where *M* is the length of the input sequence *x*, and $\bar{\text{k}}\in {\mathbb{R}}^{M}$ is a structured convolutional kernel. Leveraging Mamba's long-sequence modeling capabilities, subsequent research actively explores transferring the success of Mamba to vision, *i.e*., building a generic vision backbone purely upon SSM without attention.

### 2.2 State space model for vision tasks

In computer vision tasks, (Islam and Bertasius 2022) employ the 1D S4 model to address long-range temporal dependencies for video classification. (Nguyen et al. 2022) extend the 1D S4 framework to process multi-dimensional data, encompassing both 2D images and 3D videos. (Islam et al. 2023) integrate S4 with self-attention to construct the TranS4mer model, achieving state-of-the-art performance in movie scene detection. (Wang et al. 2023b) introduce a novel selectivity mechanism into S4, significantly enhancing performance on long-form video understanding while substantially reducing memory requirements. Jing Nathan Yan (2016) supplant attention mechanisms with a scalable SSM-based backbone capable of generating high-resolution images and processing fine-grained representations under computationally efficient constraints. (Ma et al. 2024) proposed U-Mamba, a hybrid CNN-SSM architecture designed to manage long-range dependencies in biomedical image segmentation. The aforementioned works either adapt SSMs for specialized visual applications or develop hybrid architectures combining SSMs with convolution or attention mechanisms. (Liu Y. et al. 2024) propose VMamba, which demonstrates compelling performance in visual recognition through multi-directional scanning integration and hierarchical network design. (Zhu et al. 2024) present Vision Mamba (Vim), focusing on visual sequence learning while establishing a unified representation for multimodal data. In this work, we deploy Vision Mamba as the backbone architecture for weakly supervised object localization.

## 3 Methodology

**Figure 2:**
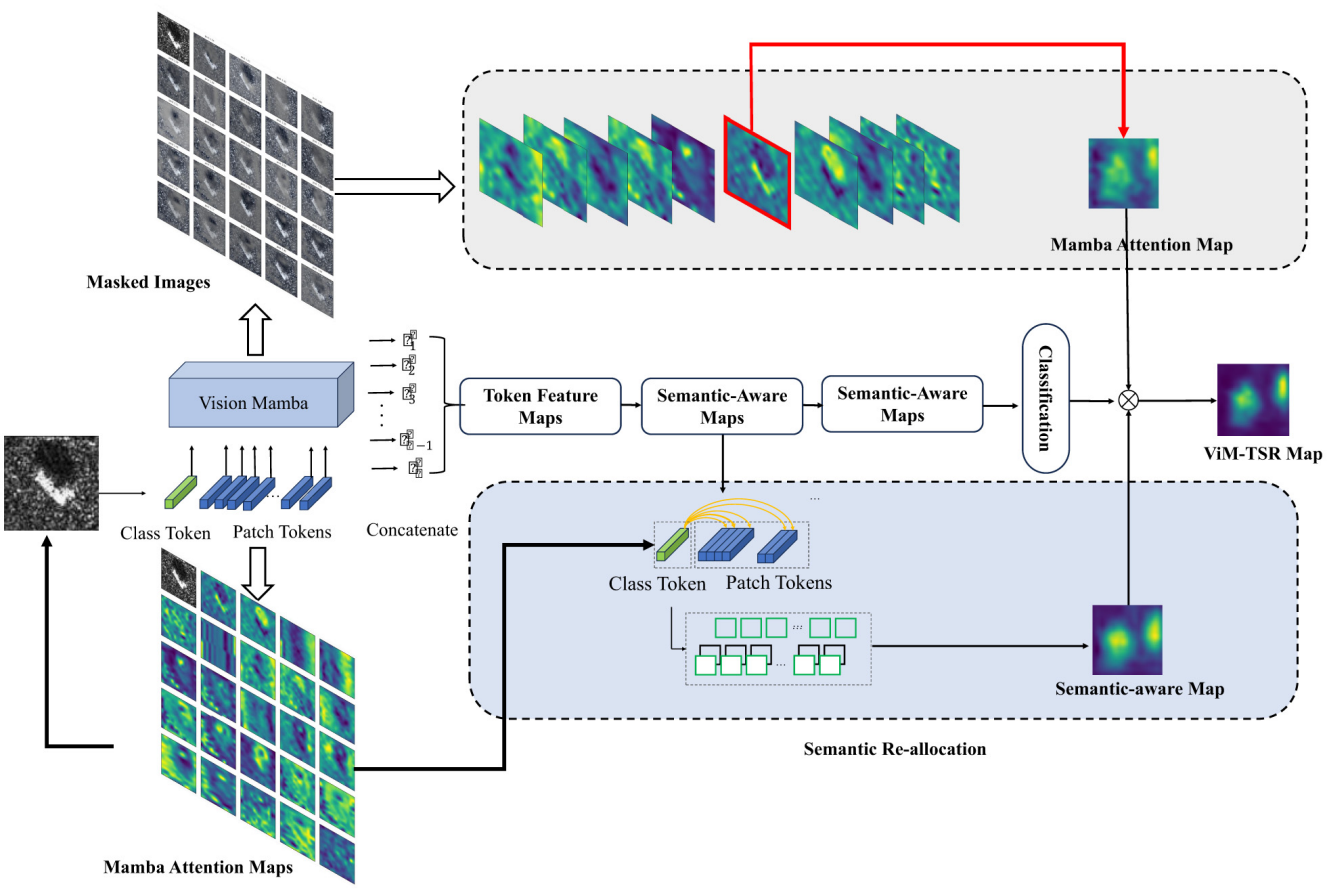
The detailed architecture of our proposed IVim for SAR image classification.

The target of IVim is to re-allocate the semantics to feature maps represented by Vim. This section begins with a description of the preliminaries of Vim, including SSM module, the mechanism of Vision Mamba and proceed to illustrate the architecture details of IVim. Then we analyze the reason why the feature maps of vanilla Vim are semantic-agnostic and subsequently introduce two modules of Ivim, i.e., TSR and TAS as shown in Figure 2.

### 3.1 Vision mamba

The vanilla Mamba model operates on one-dimensional sequences. To enable its application to visual tasks involving two-dimensional images **x** ∈ ℝ^*H*×*W*×*C*^, the image is reformatted into a sequence of flattened patches. This process yields a representation ${\text{x}}_{p}\in {\mathbb{R}}^{J\times \left(P^{2}\times C\right)}$, where *J* denotes the total number of patches and *P*^2^ is the number of pixels per patch, calculated as (*H*×*W*)/*J*.

Then, the **x**_*p*_ is linearly projected to a sequence with *D* dimension. Similar to Vision Transformer, a position embeddings ${\text{E}}_{pos}\in {\mathbb{R}}^{\left(J+1\right)\times D}$ is directly added to **x**_*p*_ to compensate spatial information of patches as Equation 5


$$\begin{array}{l} {\text{T}}_{0}=\left[t_{cls};t_{p}^{1}\text{W};t_{p}^{2}\text{W};\cdots \;,t_{p}^{J}\text{W}\right]+{\text{E}}_{pos}, \end{array}$$ (5)

where $t_{p}^{j}$ is the *j*-th patch of **x**_*p*_, **W** ∈ ℝ^(*P*^^2^*C*) × *D* is the learnable projection matrix. Inspired by ViT and BERT, we also use class token to represent the whole patch sequence, which is denoted as tcls. We then send the token sequence (**T**_*l*−1_) to the *l*-th layer of the Vim encoder, and get the output **T**_*l*_. Finally, we normalize the output class token ${\text{T}}_{L}^{0}$ and feed it to the multi-layer perceptron (MLP) head to get the final prediction $\hat{p}$, as Equation 6


$$\begin{array}{l} {\text{T}}_{l}=\text{Vim}\left({\text{T}}_{l-1}\right)+{\text{T}}_{l-1},\\ \text{f}=\text{Norm}\left({\text{T}}_{L}^{0}\right),\\ \hat{p}=\text{MLP}\left(\text{f}\right), \end{array}$$ (6)

where Vim is the proposed vision mamba block, *L* is the number of layers, and Norm is the normalization layer.

### 3.2 Interpretable vision mamba

(1) **Token semantic re-allocation (TSR)**: This mechanism transfers semantic information from the class token ($t_{0}^{L}$) to the patch tokens. The final patch token embeddings $t_{1}^{L},t_{2}^{L},\ldots ,t_{N}^{L}$ are restructured into 2D feature maps **t**^*L*^ ∈ ℝ^*D*×*w*×*h*^. A convolutional layer then processes these maps to generate a set of class-specific semantic activation maps *S*_*c*_ as Equation 7


$$\begin{array}{l} S_{c}=\underset{d}{\sum }{\text{t}}_{d}^{L}*k_{c,d} \end{array}$$ (7)

where *k*_*c, d*_ is a 3 × 3 convolutional kernel for class *c* and feature channel *d*. * denotes the convolution operation. Afterwards, IVim is trained using a novel loss function L that calculates the probability of the target class *y* based on the average activation of these maps, thereby effectively re-allocating semantic awareness to the patch tokens, which is formulated as


$$ℒ=-\log P^{'}y\;=-\log\frac{\exp(\sum_{n}S_{n,y}/N)}{\sum_{c}\exp(\sum_{n}S_{n,c}/N))}$$ (8)

where *S*_*n, c*_ denotes the semantics of *n*th patch token with respect to class *c*. By optimizing Equation 8, the semantics are reallocated to patch tokens $\left\{t_{1}^{L},t_{2}^{L},\ldots ,t_{N}^{L}\right\}$ and produce semantic-aware activation maps. After fine-tuning these convolutional kernels, they can produce feature maps that correspond with the neurons in the classification head, i.e., intuitively, each feature map matches the semantic information of its corresponding class associated, rather than vanilla Vim's feature maps that are semantic-agnostic to classes. This semantic re-allocation can provide more intuitively understanding feature maps corresponding to classes, and it is the reason why our proposed method is termed **Interpretable** Vision Mamba.

**(2) Token attention selection (TAS)**: It is difficult to generate attention map by aggregating attention matrix in TS-CAM because there is no self-attention mechanism in Vim. Specifically, Equation 4 can be extended as Equation 9


$$\begin{array}{l} \left(\begin{array}{c} y\left(0\right)\\ y\left(1\right)\\ y\left(2\right)\\ \vdots \\ y\left(N-1\right) \end{array}\right)=\left(\begin{array}{c} CBx\left(0\right)\\ CABx\left(0\right)+CBx\left(1\right)\\ CA^{2}Bx\left(0\right)+CABx\left(1\right)+CBx\left(2\right)\\ \vdots \\ CA^{\left(N-1\right)}Bx\left(0\right)+\cdots +CBx\left(N-1\right) \end{array}\right) \end{array}$$ (9)

The parameters **C**, $\bar{\text{B}}$, and $\bar{\text{A}}$ act as forgetting factors for temporal data to reduce the influence of earlier input (*x*(0), *x*(1), ⋯ , *x*(*k*−1)) on the current output, *x*(*k*). However, this mechanism is semantic-agnostic for image patches, disrupting spatial information and creating noisy activation maps, i.e., $\text{C}{\bar{\text{A}}}^{\left(k-1\right)}\bar{\text{B}}$ is semantic-agnostic. Therefore, unlike methods like TS-CAM, simply summing these maps is ineffective. To address this, we propose weighting maps by their semantic relevance. We use each layer's activation map as a mask to isolate the most salient image regions. The semantic importance of a map is then quantified by the drop in model confidence when those regions are occluded. It is necessary to assign weights to attention maps according to their semantics, so attention maps of different layers can be utilized as mask to filter the input image, i.e., only passing the most highlighted pixels while setting the rest pixels to the average value of the input image. The above procedure retains only the attention map with the highest semantic relevance to the current target class, thereby filtering out information in the attention maps that is unrelated to the target semantics and making the final heatmap more consistent with human visual intuition. This is another aspect of "interpretable" in our proposed method. Here confidence drop is adopted to measure the semantic information of the current attention map, defined as:


$$\begin{array}{l} C_{l}=\frac{P_{c}\left(x\right)-P_{c}\left(\Gamma_{W\times H}\left(A^{l}\right)⊗x\right)}{P_{c}\left(x\right)},\;l=1,2,\ldots ,L \end{array}$$ (10)

where Γ_*W*×*H*_ denotes resizing the shape of *A*^*l*^ to *W*×*H*, and ⊗ denotes Hadamard product. Equation 10 reveals that a greater *C*_*l*_ means the attention map of *l*-th layer includes more semantic information. Now, the weights of *A*^*l*^ can be defined as Equation 11


$$\begin{array}{l} \alpha^{l}=\frac{\exp\left(C_{l}\right)}{\sum_{l}^{L}\exp C_{l}} \end{array}$$ (11)

The attention map of IVim, A^*^, can be expressed by aggregating weighted *A*^*l*^, as Equation 12


$$\begin{array}{l} A^{*}=\overset{L}{\underset{l}{\sum }}\alpha^{l}A^{*} \end{array}$$ (12)

It should be clarified that there is indeed no standard self-attention matrices in Mamaba. The matrices *CB*, *CAB*,..., *CA*^*k*−1^*B* in vanilla Mamba are actually regarded as “forgetting factors” to assign smaller weights to former inputs and bigger weights to later inputs for one dimensional temporal sequences. However, image input does not have temporal correlations among pixels, thus there we just use attention matrix to denote *CB*, *CAB*,..., *CA*^*k*−1^*B*. It is not dervied from the convolutional kernel, becuase only TSR utilizes the convolutional kernel to re-allocate the smantics to feature maps. *CB*, *CAB*,..., *CA*^*k*−1^*B* are directed extracted from the Mamba while forward inference.

**Figure 3:**
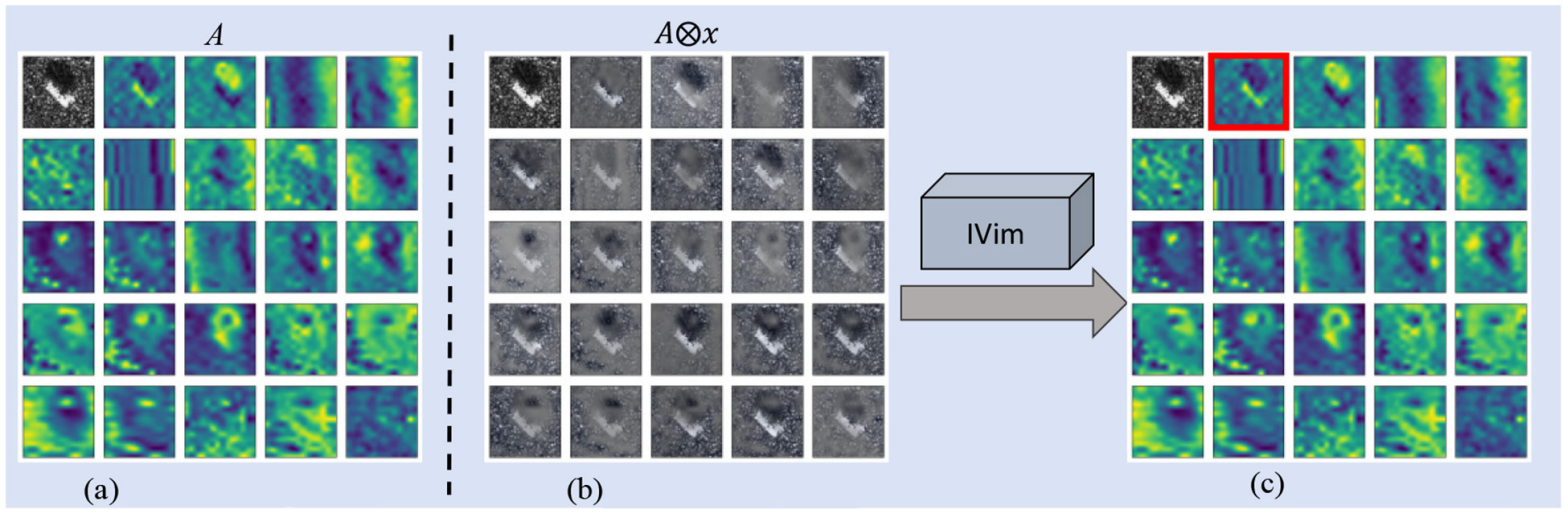
The visualization of Token Attention Selection module. **(a)** Attention maps. **(b)** Masked images. **(c)** Selection result.

**(3) Saliency map aggregation**: Finally, a saliency heatmap for interpretation can be aggregated by calculating the Hadamard product of A^*^ and *S*_*c*_, as shown in Figure 3, which can be expressed as Equation 13


$$\begin{array}{l} H_{c}=\Gamma_{W\times H}\left(A^{*}\right)⊗S_{c} \end{array}$$ (13)

*H*_*c*_ is the saliency map where most highlighted regions reflect the most discriminative pixels for classification. It is worth noting that this step is unnecessary to forge IVim, while it is only used to provide a saliency map to visualize the interpretabilty of IVim in SAR image classification.

## 4 Results

### 4.1 Experiment settings

We adopt a widely-used benchmark SAR dataset, MSTAR, to demonstrate the validity of our proposed IVim. MSTAR includes 2536 SAR images of 10 classes of vehicles for training and 2, 636 for validation. It is worth noting that the original SAR images are gray-scale; however, to avoid modification of the parameters of Vim, all the SAR images are transformed into pseudo-RGB images (reduplicate the monochromatic image in three channels). The MSTAR images are uniformally resized to 224 × 224. The backbone Vim is trained by AdamW with a momentum of 0.9, a total batch size of 1024, and a weight decay of 0.05 to optimize models. The Vim is pretrained on ImageNet-1k for 300 epochs using a cosine schedule, 10^−3^ initial learning rate. We use a convolutional layer with 3 × 3 kernel followed by the classifying head to re-allocate semantics to feature maps. The learnable parameters of above convolutional layer are fine-tuned on MSTAR by 50 epochs. Additionally, we assert the rationale for the choice of above experimental parameters settings. The optimizer, batch size, weight decay of the backbone are identical to the parameter setting in (Zhu et al. 2024). The kernerl size is set to 3 × 3 to ensure a balance between global feature representation and detailed contextual perception. It is worthnoting that all layers are frozen except these convoutional kernerls when optimizing Equation 4. Experiments are performed on a NVIDIA RTX 4090 GPU.

### 4.2 Results analysis

**Classification**: Table 1 presents the comparison of classification accuracy of different deep neural networks. It is evident that the proposed IVim obtains the superior classification accuracy to other models. Although IVim is not designed solely to improve classification performance, Table 1 demonstrate sthat reassigning semantics to the deeper features of Mamba can provide more information for the classification layer, ultimately enhancing the classification accuracy. It should be clarified that classic models are usually regarded as capable enough for MSTAR classification becuase the number of samples are small and the images are cropped in center with pure background. However, it is still necessary to compare most state-of-the-art model to demonstrate the superiority of the baseline Mamba and our proposed Ivim. Hence, we particularly compared a recent a SOTA architecture i.e., DisCo DETR) for object classification proposed in Table 1.

**Table 1:** Comparison of classification accuracy of our proposed IVim and other models in SAR images.

Model	Ratio of training samples				
	10%	30%	50%	70%	100%
MobileNetV2	71.84 ± 0.979	96.05 ± 0.355	97.68 ± 0.330	98.94 ± 0.155	99.11 ± 0.199
ASIR-Ne	58.23 ± 3.737	88.26 ± 3.101	95.11 ± 0.994	97.59 ± 0.363	99.15 ± 0.033
ResNet18	86.21 ± 0.543	97.01 ± 0.284	98.47 ± 0.286	99.11 ± 0.056	99.43 ± 0.066
ResNet34	87.32 ± 0.703	97.49 ± 0.259	98.57 ± 0.100	99.12 ± 0.139	99.42 ± 0.086
RepVGG	71.24 ± 1.554	93.13 ± 1.344	97.78 ± 0.467	98.53 ± 0.433	99.39 ± 0.238
SFAS	79.77 ± 2.136	95.27 ± 3.515	98.38 ± 0.181	98.99 ± 0.092	99.45 ± 0.037
MobileViT	72.03 ± 0.398	95.88 ± 0.622	98.66 ± 0.317	99.13 ± 0.181	99.46 ± 0.159
DenseNet	88.61 ± 0.837	97.98 ± 0.182	98.90 ± 0.280	99.54 ± 0.075	99.65 ± 0.118
DisCo DETR	89.21 ± 0.125	97.45 ± 0.042	98.25 ± 0.140	99.21 ± 0.161	99.45 ± 0.124
**Ivim**	**89.94** **±** **0.274**	**97.33** **±** **0.092**	**99.07** **±** **0.141**	**99.57** **±** **0.045**	**99.62** **±** **0.018**

Bold values indicate the method with best performance.

**Interpretability**: To analyze the interpretability of IVim quantitatively, we implemented a perturbation strategy which occludes most highlighted or weakest pixels in the input SAR image to check the change of model's prediction. Specifically, perturbation can be categorized into an “occlusion game” and a “conservation game”. The occlusion game measures the importance of those highlighted pixels for classification when they are occluded. In the occlusion game, the input image *I* is masked by the heatmap, as Equation 14


$$\begin{array}{l} Ǐ=I⊗M^{Occlusion} \end{array}$$ (14)

where Ǐ denotes perturbed image and *M*^*CAM*^ denotes a binary-value mask defined as Equation 15


$$\left\{ \begin{array}{l} M_{ij}^{Occlusion}=0,\;\;\;H_{ij}^{CAM}\geq 0.8,\\ M_{ij}^{Occlusion}=1,\;\;\;\text{otherwise}, \end{array}\right.$$ (15)

Here $H_{ij}^{Occlusion}$ has been normalized to [0, 1]. This means that in mask *M*^*Occlusion*^, the elements corresponding to the top 20% value of the heatmap are set to 0, while the rest are equal to the heatmap. Then, we compute the divergence of the class confidence (the output of the softmax layer) between the original image and the perturbed image, as Equation 16


$$\begin{array}{l} confidence\text{\_}drop\left(I,Ǐ\right)=\frac{S_{c}\left(I\right)-S_{c}\left(Ǐ\right)}{S_{c}\left(I\right)} \end{array}$$ (16)

In the occlusion game, a low value of *confidence*_*drop* means that more discriminative pixels of the object are covered by heatmaps. Nevertheless, the salient regions sometimes almost cover the entire image, which can also lead to a high value of *class*_*score* in the occlusion test in Table 2. In this case, it is necessary to introduce "conservation game". The only difference between the conservation game and the occlusion game is the mask *M*^*Conservation*^ formulation, defined as Equation 17


$$\begin{array}{l} M^{Conservation}=U-M^{Occlusion} \end{array}$$ (17)

where *U* is a matrix ∀*i, j*, *U*_*ij*_ = 1. The results of both occlusion and conservation game are presented in Table 2. Table 2 reveals that IVim can produce the most discriminative heatmaps for interpreting Mamba's feature representation mechanism. It should be noted that gradient-based methods are better than gradient-free methods in occlusion game but worse than those in conservation methods. It is because gradient-based methods tend to cover the background region out of the object's profile.

**Table 2:** Comparison of interpretability of our proposed IVim and other interpretation methods.

Method	Interpretation capability	
	Occlusion	Conservation
Grad	0.85	0.25
Grad++	0.88	0.21
XGrad	0.79	0.28
Ablation	0.82	0.24
Score	0.90	0.24
IVim	0.94	0.18

**Efficiency Analysis**: Table 3 compares the parameter complexity of our proposed IVim and other models. Obviously, IVim achieves the best classification performance with the smallest scale of paremeters (67.8% lower than that of ResNet-34). We also found that IVim occupies 4.6GB CUDA memory with 224 × 224 and only 11.2GB CUDA memory with 1200 × 1200 while DeiT-Ti occupies more than 70GB CUDA memory.

**Table 3:** Comparison of efficiency of different WSOL backbones.

Backbones	Image size	Params (M)	Top-1 *Cls. Acc* (*%*)
VGG16	224^2^	138.3	94.40
ResNet34	224^2^	21.8	99.42
Deit-S	224^2^	25.1	99.53
IVim (Vim-T)	224^2^	7	99.62

### 4.3 Ablation study

**Figure 4:**
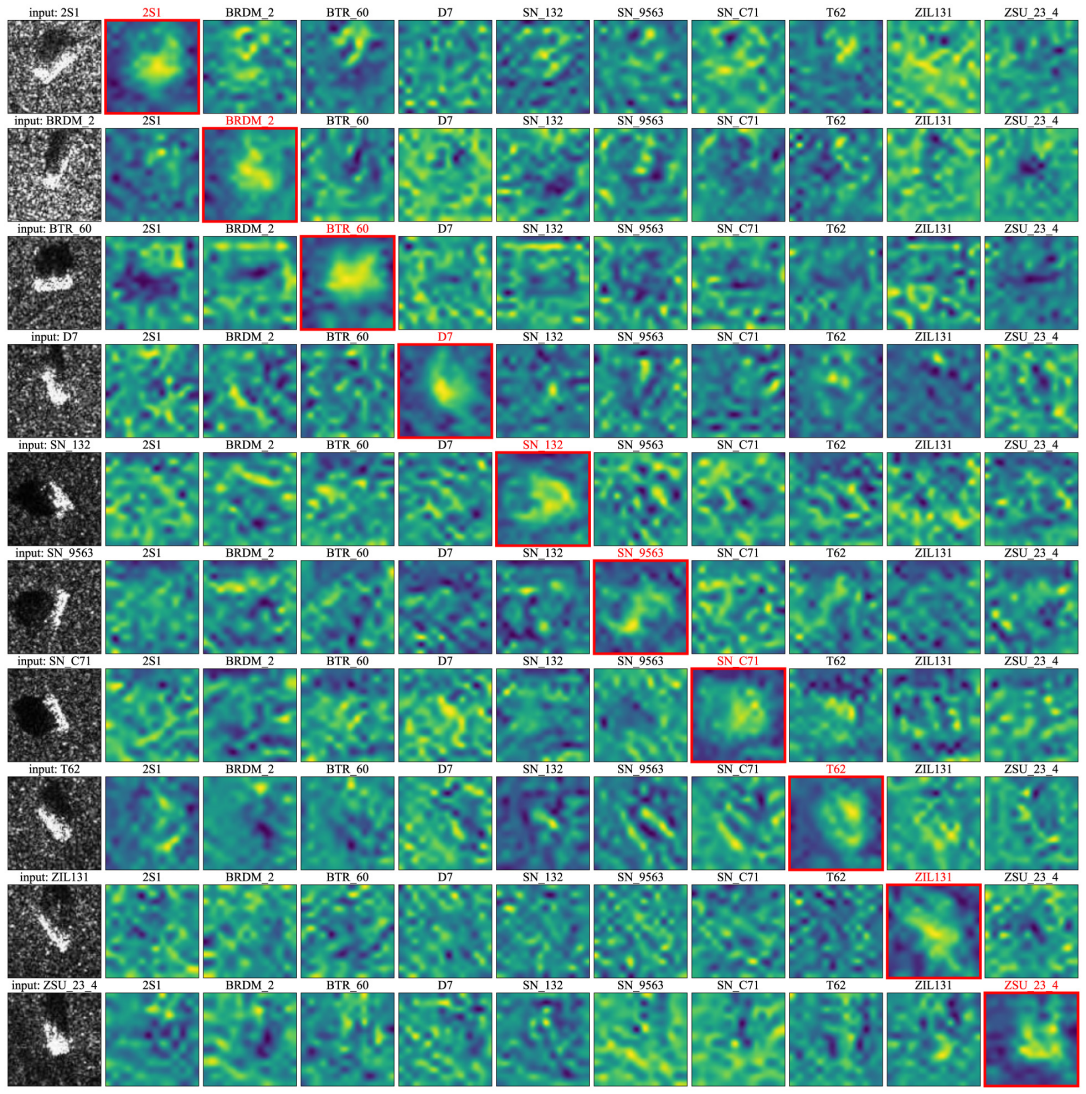
Visualizing the semantics of *S*_*c*_ for different classes (best view ed in color).

**Mechanism of TSR**: Figure 4 shows the mechanism of semantics re-allocation, *i.e*., *S*_*c*_ for different classes. The first column shows input images of 10 classes, and the second to the eleventh column presents semantic-aware feature maps corresponding to different classes, *S*_*c*_, where the *Sc* corresponding to the current class is marked by a red square. Figure 4 vividly shows that the semantic information is allocated to the feature map corresponding to the class of input image, while other feature maps are completely semantic-agnostic. It demonstrates TSR module effectively introduce the semantics by optimizing the proposed loss function, i.e., *S*_*c*_. Table 4 further presents quantitative ablation study results. It is clear from Table that TSR and TSA collaboratively work to increase the attention concentration solely on regions relevant to the obejct semantics. When TSR module is removed, TSA even produce slight lower classification performance (99.48%) than vanilla baseline (99.53%) because TSA can only select semantic-agnostic feature maps without smantic re-allocation by TSR.

**Table 4:** Ablation of Semantic-aware actiavtion and semantic-agnostic actiavtion.

TSR	TSA	Top-1 *Loc. Acc*
		99.53
✓		99.75
	✓	99.48
✓	✓	99.62

**Figure 5:**
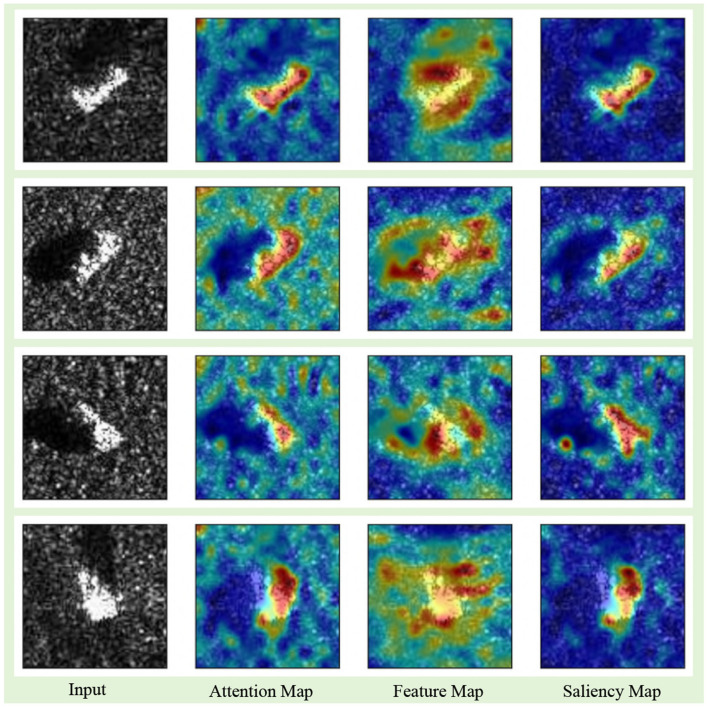
The comparison of attention map of vanilla Vim, Feature Map of IVim, and the saliency heatmap of IVim.

**Mechanism of TAS**: Figure 5 shows the mechanism of TAS module. The first column shows the input images of 10 classes, the second column presents the attention maps producde by TAS, the third column presents the feature maps produced by TSR, and the last columns shows the aggregated saliency heatmaps. It is clear from Figure 5 that attention maps can locate the object profile well, but some scatters are randomly distributed around the most highlighted region. In this scenario, the element-wise multiplication of *a* and *S*_*c*_ effectively counteracts their respective limitations and synergizes their strengths, forming a heatmap that interprets the feature representation mechanism of Mamba model.

## 5 Conclusion

We proposed an interpretable vision Mamba (IVim) for SAR image classification. IVim fully takes the advantages of the long-range feature dependency and low occupancy on GPU memory of Vim. To solve the the semantic agnostic issue of the feature maps, we proposed TSR module to re-allocate semantic information for feature maps, enabling each of them to be aware of object class. Furthermore, we proposed the TSA module, which employs a dynamic weighting strategy to selectively combine attention matrices from different layers of the Mamba, thereby generating semantically interpretable attention maps. Experiments on the MSTAR datasets demonstrate that IVim conspicuously improved the interpretability of feature maps in Mamba block, in striking contrast with its other counterparts.

## Data Availability

Publicly available datasets were analyzed in this study. This data can be found here: https://www.sdms.afrl.af.mil/index.php?collection=mstar.
